# The Role of Hsp27 in Chemotherapy Resistance

**DOI:** 10.3390/biomedicines10040897

**Published:** 2022-04-14

**Authors:** Marios Lampros, Nikolaos Vlachos, Spyridon Voulgaris, George A. Alexiou

**Affiliations:** Department of Neurosurgery, University Hospital of Ioannina, St. Niarhou Avenue, 45500 Ioannina, Greece; marioslampros@gmail.com (M.L.); nickosvlachos826@gmail.com (N.V.); svoulg@otenet.gr (S.V.)

**Keywords:** Hsp27, cancer, chemotherapy

## Abstract

Heat shock protein (Hsp)-27 is a small-sized, ATP-independent, chaperone molecule that is overexpressed under conditions of cellular stress such as oxidative stress and heat shock, and protects proteins from unfolding, thus facilitating proteostasis and cellular survival. Despite its protective role in normal cell physiology, Hsp27 overexpression in various cancer cell lines is implicated in tumor initiation, progression, and metastasis through various mechanisms, including modulation of the SWH pathway, inhibition of apoptosis, promotion of EMT, adaptation of CSCs in the tumor microenvironment and induction of angiogenesis. Investigation of the role of Hsp27 in the resistance of various cancer cell types against doxorubicin, herceptin/trastuzumab, gemcitabine, 5-FU, temozolomide, and paclitaxel suggested that Hsp27 overexpression promotes cancer cell survival against the above-mentioned chemotherapeutic agents. Conversely, Hsp27 inhibition increased the efficacy of those chemotherapy drugs, both in vitro and in vivo. Although numerous signaling pathways and molecular mechanisms were implicated in that chemotherapy resistance, Hsp27 most commonly contributed to the upregulation of Akt/mTOR signaling cascade and inactivation of p53, thus inhibiting the chemotherapy-mediated induction of apoptosis. Blockage of Hsp27 could enhance the cytotoxic effect of well-established chemotherapeutic drugs, especially in difficult-to-treat cancer types, ultimately improving patients’ outcomes.

## 1. Introduction

Heat shock protein (Hsp) 27 is an ATP-independent, small-sized (approximately 27 kDalton) chaperone molecule and a member of the small Hsp family [1,2,3]. It is a single copy gene covering approximately 2.2 kb transcripts, has three coding exons responsible for producing a 205 amino acid protein, and is encoded by the HSPB1 gene [4,5]. While it was thought to be activated only during heat shock conditions when it would assist in the refolding of damaged or misfolded proteins [6], recent studies suggest that Hsp27 is overexpressed after exposure to several other cellular stressors, including hypoxia, oxidative stress, infections, and ultraviolet radiation, where, through different molecular mechanisms, it facilitates proteostasis and cell survival [5,7,8]. Hsp27 molecules can be found in cells as small dimers, probably the active units of Hsp27’s tetramers, or as large oligomers formed by multiple dimers [1,2]. Regarding its structure, Hsp27 is composed of the N-terminal domain, the α-crystallin domain, and the C-terminal domain. The N-terminal domain accommodates a poorly-conserved WDPF motif necessary for large oligomerization, whereas the C-terminal end contains the highly-conserved α-crystallin motif essential for small oligomerization [9,10,11,12,13]. The extent of oligomerization is primarily driven by the dynamic phosphorylation of Hsp27, which is regulated by cell conditions [14]. Under normal cell conditions, Hsp27 remains unphosphorylated with the ability to form large oligomers up to 1000 kDa [15] whereas, under stress, Hsp27 is phosphorylated, resulting in the formation of small oligomers (often dimers or tetramers) lacking chaperone activity [6,16,17]. Phosphorylation of Hsp27 can occur on three different serine (Ser) residues (Ser-15, Ser-78, Ser-82) and one Threonine (Thr) residue (Thr-143). However, studies have shown that only Ser-78 and Ser-82 are profoundly involved in that phosphorylation while Ser-15 has only minor participation [18,19]. Numerous kinases are associated with the Hsp27 phosphorylation process. P38 mitogen-activated protein kinase (MAPK), protein kinase C (PKC), and activated protein kinase 2 (MK2) and 3 (MK3) can phosphorylate Ser-15. Ribosomal S6 kinase (p70RSK), PKC, protein kinase G, and MK2, MK3, can phosphorylate Ser-78 while Ser-82 can be phosphorylated by MK2, MK3, p70RSK, protein kinase B (PKB), and protein kinase D (PKD) [18,19,20]. The phosphorylation of Hsp27 leads to altered molecular functions, including interaction with intermediate filaments and subsequent regulation of cell migration, modulation of cell cycle progression through inhibition of the MEK/ERK signaling pathway or interaction with p53, and inhibition of apoptosis [5,7,8,21,22,23]. Hsp27 is overexpressed in various cancer cell types, and interestingly, these cancer types have displayed resistance to chemotherapeutic agents. The subsequent tumor progression and metastatic potential have been attributed to the increased Hsp27 expression or Hsp27 phosphorylation [1,2,22,23]. In our review, by focusing on in vitro studies in various cancer cell lines, we attempted to elucidate whether there is indeed an association between Hsp27 overexpression and chemotherapy resistance, as in such a case, inhibition of Hsp27 could open the door to newer, more effective anticancer treatment regimens.

## 2. The Role of Hsp27 in Cancer

Hsp27 overexpression has been observed in numerous cancer types, including gliomas, medulloblastomas breast, ovarian, prostate, non-small lung cancer, and hepatocellular carcinoma [1,3,24,25,26,27,28]. Although Hsp27 levels have been correlated with cancer patients’ clinicopathological and prognostic features, with higher expression associated with more advanced disease stage and poorer prognosis, until today, Hsp27 has not officially been established as a cancer biomarker in everyday clinical practice [2,29,30,31,32,33,34]. Hsp27 affects tumor initiation, invasiveness, and metastasis through regulating different molecular pathways.

### 2.1. Modulation of the “Salvador-Warts-Hippo” (SWH) Pathway

**Figure 1:**
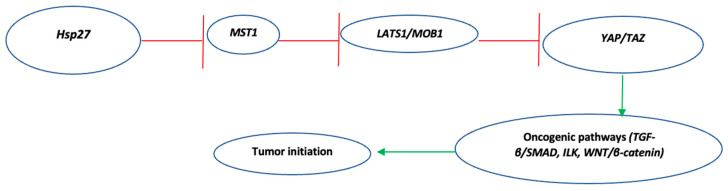
Summary of Hsp27-mediated modulation of the Salvador-Warts-Hippo (SWH) pathway, eventually leading to tumorigenesis. Green indicates activation, Red indicates inhibition. Key: MST1, macrophage stimulating 1; LATS1, large tumor suppressor kinase 1; MOB1, MOB Kinase activator 1A; YAP, Yes-associated protein; TAZ, PDZ-binding motif.

Vahid et al., in their in vitro studies using breast (MDA-MB-453), lung (A549), and prostate (PC3) cancer cell lines, described how Hsp27 modulates the Hippo tumor suppressor pathway [35]. The “Salvador-Warts-Hippo” (SWH) pathway represents a highly conserved signaling pathway associated–among others–with the control of organ growth and cell proliferation, whose downregulation correlates with tumor initiation, progression, and poor patient outcome across different cancer types [36,37]. In normal cells, this pathway initiates with the activation of mammalian STE20-like protein kinases 1 and 2 (MST1/2), which through phosphorylation of large tumor suppressor kinases 1 and 2 (LATS1/2) and adaptor proteins Mps one binder kinase activator 1 (MOB1), results in the phosphorylation of the two transcription factors Yes-associated protein (YAP)/transcriptional co-activator with PDZ-binding motif (TAZ). Therefore, the YAP/TAZ complex remains in the cytoplasm, along with 14.3.3 proteins [37]. However, according to the authors’ in vitro studies, in cancer cells, the overexpressed Hsp27 forms a complex with endogenous MST1, eventually leading to its ubiquitin-mediated degradation. Thus, LATS1 and MOB1 remain unphosphorylated, as do the two transcriptional co-activators YAP/TAZ, which translocate to the nucleus, promoting oncogenic cascades including TGF-β/SMAD, WNT/β-Catenin, and ILK pathways [35,38,39,40]. The role of Hsp27 in promoting tumorigenesis via modulation of the SWH pathway is summarized in Figure 1.

### 2.2. Promotion of Epithelial-Mesenchymal Transition (EMT)

Two studies investigated how Hsp27 facilitated epithelial to mesenchymal transition (EMT) in prostate cancer cells in vitro [41,42]. EMT plays a pivotal role in cancer cell dissemination to secondary sites by enabling malignant epithelial cells to acquire mesenchymal characteristics with defined morphology, protein expression, and gene signatures. Several different signaling pathways are involved in this complex procedure, with TGF-β being the most popular one. TGF-β binds to TGF-β receptor type II which, in turn, phosphorylates TGF-β receptor type I, promoting its kinase activity either through Smad-dependent or Smad-independent signaling cascades. In the former case, the Smad2/3 complex, after its phosphorylation, binds to Smad4 and the complex then translocates to the nucleus, while in the Smad-independent signaling cascades, a number of downstream molecular pathways are involved, including Wnt/β-catenin, PI3K/Akt, JNK, ERK, p38/MAPK, and Ras small GTPases. Both Smad-dependent and Smad-independent signaling pathways interact with various transcriptional repressors in the nucleus, such as Twist 1/2 and SNAIL (SNAI) 1/2, LEF-1 and zinc finger E-box binding (ZEB)1/2, eventually leading to cytoskeletal changes such as loss of E-cadherin [43,44]. Other signaling pathways which play a significant role in EMT include Notch, Wnt/β-catenin, and Hedgehog signaling pathways. Their role in EMT promotion has been described in previous reviews [44,45].

**Figure 2:**
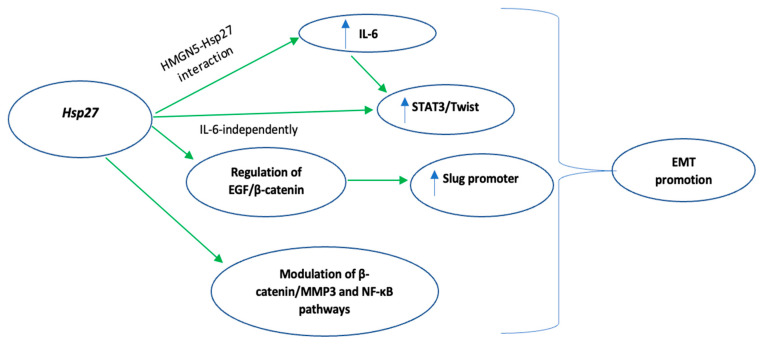
Summary of the molecular mechanisms of Hsp27-mediated promotion of Epithelial-mesenchymal transition (EMT). Green indicates activation. Key: EMT, epithelial to mesenchymal transition; EGF, epidermal growth factor; STAT3, signal transducer and activator of transcription 3; NF-kb, nuclear factor kappa-light-chain-enhancer of activated B cells; IL-6, interleukin 6.

Regarding the role of Hsp27 in EMT regulation [46,47], Shiota et al. found that Hsp27 induces STAT3 phosphorylation, either through IL-6 mediation or independently, and the subsequent STAT3 nuclear translocation and binding to the TWIST promoter, enhances EMT [41]. Hsp27-mediated upregulation of the same signaling pathway (IL-6/STAT3/Twist) promoted EMT in four different bladder cancer cell lines. Of note, the upregulation of the above-mentioned signaling pathway was facilitated by Hsp27 interaction with High-mobility group nucleosome-binding domain 5 (HMGN5), a tumorigenesis-related molecule [48]. Cordonnier et al. [42], using the same prostate cancer cell line (LNCaP) as Shiota et al. [41] suggested that Hsp27 overexpression, through regulation of the EGF/β-catenin cascade, supported β-catenin’s nuclear translocation and its subsequent binding to the SNAIL 2 (Slug) promoter, eventually promoting EMT [42,49]. Moreover, four different studies [50,51,52,53] supported the role of Hsp27 in facilitating TGF-β1-mediated EMT either by protecting SNAIL from proteasome degradation [51] or by upregulating TGF-β1/p38 pathway [53]. Fang et al., in their in vitro study, using pancreatic ductal adenocarcinoma cell lines, highlighted the Hsp27-induced modulation of the β-catenin/MMP3 signaling cascade [54], a crucial pathway of the EMT, while Zhu et al. proposed that Hsp27 promotes EMT via upregulation of the NF-kB signaling pathway [55]. The molecular mechanisms by which Hsp27 promotes EMT are summarized in Figure 2.

### 2.3. Adaptation of CSCs to Stresses of the Tumor Microenvironment

**Figure 3:**
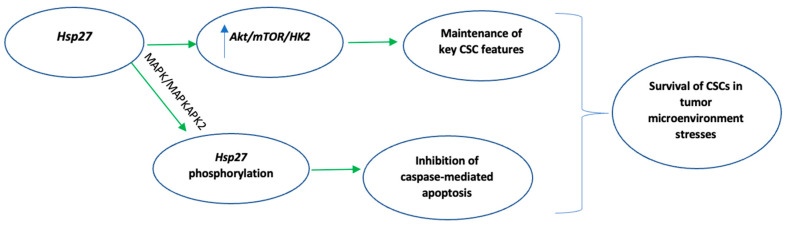
Summary of Hsp27-mediated molecular mechanisms which enable cancer stem cells to survive tumor microenvironment stresses. Green indicates activation. Key: CSC, cancer stem cells; PI3, phosphatidylinositol-3-kinase; mTOR, mammalian target of rapamycin.

In human esophageal squamous cancer cell line (CE81T and TE1) cultures, a subpopulation demonstrated cancer stem-cell (CSC) properties, including increased aldehyde dehydrogenase (ALDH) activity, a tumor stem cell-associated marker in many cancer types [56]. According to the CSC hypothesis, CSCs contribute to the initiation and progression of various malignancies [57,58]. Liu et al. reported that in the subpopulation of esophageal CSCs, these CSCs demonstrated overexpression of Hsp27 that helped them retain key CSC features, including increased glycolysis and oxidative phosphorylation. These CSC features were attributed to the activation of the AKT/mTOR/HK2 pathway by direct interaction of Hsp27 with AKT, ultimately resulting in increased HK2 expression, which according to the authors, is necessary for esophageal CSCs to maintain the afore-mentioned distinct metabolic phenotype [56]. Lin et al. described that CD133+ CSCs from four different cancer types (colorectal, brain, lung, and oral cancer) managed to survive hypoxia and serum depletion-mediated apoptosis through upregulation of the p38 MAPK/MAPKAPK2 pathway, phosphorylation of Hsp27 and inhibition of caspase-mediated apoptosis [59]. Activation of the latter pathway (p38 MAPK/MAPKAPK2/Hsp27) enabled CD133+ colon CSCs to resist antiangiogenesis-induced apoptosis, according to Lin et al. [60]. The molecular mechanisms by which Hsp27 results in the adaptation of CSCs to stresses of tumor microenviroment are summarized in Figure 3.

### 2.4. Induction of Angiogenesis

Hsp27 in the peripheral blood, through indirect interaction with toll-like receptor 3 (TLR3) and subsequent NF-κB activation, increased the secretion of vascular endothelial growth factor (VEGF) and activated VEGF receptor type 2 in breast cancer cells, ultimately promoting angiogenesis [61,62]. Straume et al. suggested that suppression of Hsp27 in breast cancer cells led to a decrease in the expression of VEGF-A, VEGF-C, and basic fibroblast growth factor through downregulation of NF-κB and STAT3, thus highlighting the Hsp27’s proangiogenic role [63].

### 2.5. Inhibition of Apoptosis

Numerous studies have highlighted the inhibitory role of Hsp27 in both caspase-dependent and caspase-independent apoptotic pathways [62,64,65,66,67,68,69,70,71,72]. With respect to the inhibition of caspase-mediated apoptosis, Hsp27 either interacts directly with caspase-3 or cytochrome c, thus inactivating them [64,65], or upregulates PI3-kinase which activates Akt, and the subsequent Akt-induced Akt/Bax interaction eventually blocks Bax translocation to the mitochondria [66,70,71]. In myeloma cell lines, Hsp27 was found to interact directly with Smac (second mitochondria-derived activator of caspase), therefore inhibiting the activation of caspase-3 [65,67]. Hsp27 also inactivates p53, which is known to regulate p21, a cyclin-dependent kinase inhibitor that modulates cell cycle progression. This leads to increased p21 phosphorylation, translocation to the cytoplasm, and eventually, increased cell survival [62,70,71].

**Figure 4:**
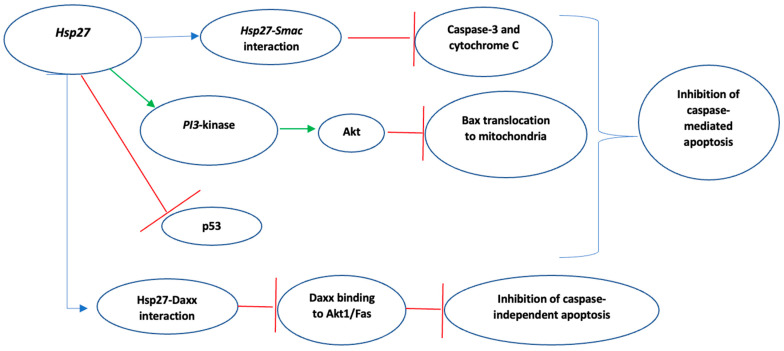
Summary of Hsp27-mediated inhibition of apoptosis. Green indicates activation, Red indicates inhibition. Key: PI3, phosphatidylinositol-3-kinase.

Moreover, Hsp27 affects caspase-independent apoptotic pathways via direct interaction with Daxx (death domain-associated protein 6), thus blocking Daxx binding to the Akt1 (apoptosis-signal-regulated kinase 1)/Fas (regulator of cell death) complex and ultimately inhibiting the Akt1/Fas-mediated apoptosis [62,64,71,72,73]. The molecular mechanisms by which Hps27 inhibits caspase-dependent and caspase-independent apoptosis are summarized in Figure 4.

## 3. The Role of Hsp27 in Chemotherapy Resistance

### 3.1. Doxorubicin

Doxorubicin is an efficient chemotherapy agent utilized in multiple cancer types such as breast, lung, lymphomas, and gastrointestinal cancers. Its anticancer effects are primarily mediated through intercalation into DNA and inhibition of topoisomerase II, thus promoting a G2/M cell cycle arrest. Doxorubicin also induces oxidative stress in cancer cells via a complex mechanism that involves its oxidation to semiquinone, an unstable metabolite, leading to the formation of free radicals. Moreover, doxorubicin upregulates Fas ligands. The latter two mechanisms result in mitochondrial dysfunction, caspase activation, and a subsequent trigger of apoptotic pathways [74]. In 1992, Ciocca et al., in their in vitro study, found that the increased expression levels of Hsp27 and Hsp70 in breast cancer cell lines were related to an increased chemotherapy resistance against doxorubicin, and this was independent of the heat shock-induced overexpression of the P-gp, the principal molecule involved in multidrug resistance (MDR). The authors utilized two breast cancer cell lines, the MCF-7/BK cell line in which Hsp27 is constitutively expressed while the expression levels do not significantly fluctuate after heat shock, and the MDA-MB-231 cell line that has a baseline low Hsp27 expression, that increases considerably under heat shock conditions. At 37 °C, the MCF-7/BK line was seven times more resistant to doxorubicin compared to MDA-MB-231. After heat shock, in the MDA-MB-231 cell line, the level of resistance was 17 times higher compared to baseline, whereas in the MCF-7/Bk cell line, it was three times higher compared to baseline. This effect was not observed with other commonly utilized chemotherapy drugs such as cisplatin, actinomycin, methotrexate, and 5-FU. Finally, the authors reported that the doxorubicin resistance was not significantly increased in the multidrug-resistant MDA-A1R cell line after exposure to heat shock, probably indicating that the MDR was not mediated via an Hsp27 mediated mechanism [75]. A similar effect in Hsp27-transfected MDA-MB-231 cells after treatment with doxorubicin was reported by Oesterreich et al., but additionally, the authors mentioned that the overexpression of Hsp27 in these cells enhances the anchorage-dependent and anchorage-independent growth in vitro [76]. Additionally, a cytoprotective effect of Hsp27 against doxorubicin, via an MDR-independent mechanism, was also observed by another in vitro study, in which ten thermoresistant Chinese hamster cell lines were transfected with a plasmid containing the whole gene of human Hsp27 [77] as well as in a study in which HT-29 human colon cancer cell lines were transfected with the Hsp27 gene [78]. In the former case, Huot et al. reported a statistically significant, positive correlation between the expression levels of Hsp27 and cellular survival after exposure to doxorubicin, while the expression levels of P-gp or the intracellular concentrations of doxorubicin were similar between different clones with different expression levels of Hsp27 or with different intracellular concentrations of doxorubicin [77]. Garrido et al. observed that although the expression levels of Hsp27 in HT-29 or CaCo2 human colon cancer cell line were correlated with increased resistance against doxorubicin, this was reversed when the cells were previously exposed to cisplatin. The cisplatin-induced Hsp27 overexpressing cells had, paradoxically, decreased chemoresistance to doxorubicin. Cisplatin is a chemotherapy agent that cross-links the purine bases of DNA and affects the cell cycle, inducing a cell arrest at the S phase. In that phase of the cell cycle, the levels of topoisomerase II, the main anthracycline target, are increased, and hence the cytoprotective effect of Hsp27 against doxorubicin could be covered by the increased targeting of topoisomerase II [78]. In another study, Hansen et al. found that the expression of topoisomerase II was lower in Hsp27 transfected MDA-MB-231 breast cancer cells than in controls and inhibited the doxorubicin-induced apoptosis, suggesting a potential mechanism explaining the Hsp27-mediated cancer resistance. Specifically, the authors observed that in Hsp27-overexpressing cells, the basal expression of topoisomerase IIa and topoisomerase IIb was significantly (*p* < 0.05) decreased compared to control. After treatment with 0.1 mg/dL doxorubicin, the expression levels were increased in both cell lines, but overall, a reduction–approximately twofold–of topoisomerase IIa and IIb was observed in the Hsp27-overexpressing cell line. Although the latter indicates that Hsp27 decreases the expression levels of p53, the exact mechanism of this Hsp27-mediated reduction in topoisomerase II expression remains unclear [79]. However, it has been suggested that p53 can stimulate the activation of topoisomerase II [80,81]. Doxorubicin can induce the formation of ROS, which in turn, stimulates the activation of p53. Hsp27 is an inhibitor of p53 activation. Hence, in Hsp27-overexpressing cells, the Hsp27-mediated inhibition of p53 could explain the decrease in the expression levels of topoisomerases II. However, the latter mechanism is hypothetical, and future studies should be conducted to clarify the exact mechanism of Hsp27-mediated inhibition. Interestingly, the authors did not observe any differences in the levels of several known pro-apoptotic and anti-apoptotic proteins of the intrinsic apoptotic pathway, such as bak and Bcl-2. Moreover, the specific cell line has a known mutated p-53 gene. Thus, the authors concluded that the regulation of the doxorubicin-induced apoptosis is probably mediated by a mechanism involving the suppression of apoptotic genes [82]. Paclitaxel, a tubulin target, has been suggested to decrease the expression levels of Hsp27 and hence could be utilized to reduce the doxorubicin resistance in Hsp27 overexpressing tumor cells [83,84]. In their in vitro study, Shi et al. utilized three distinct breast cancer cell lines, the Hsp27-overexpressing MCF-7 and MDA-MB-435, and the low-expressing MDA-MB-231 cell line. Paclitaxel downregulated the expression levels of Hsp27 only in the Hsp27-overexpressing cell lines, while it only had a minimal effect on the MDA-MB-231 line. This downregulation was combined with an increase in the expression levels of topoisomerases IIa and IIb in the MDA-MB-435 and MCF-7 cell lines, and paclitaxel-doxorubicin administration was more effective compared to doxorubicin-paclitaxel administration, as the former yielded a higher rate of apoptosis. However, the exact mechanism behind the paclitaxel-induced Hsp27 downregulation was not described [82].

Even though the protective effect of Hsp27 in doxorubicin-mediated cell death has been described by several studies, only limited data exist regarding the exact mechanism of cytoprotection. O’Callaghan-Sunol et al., in their in vitro study, noted that Hsp27 induces the inhibition of p53 and the subsequent inactivation of p21, a cyclin-dependent kinase inhibitor, ultimately resulting in inhibition of apoptosis and promotion of cellular survival [85]. The specific mechanism of Hsp27-induced inhibition of p53 was not explored in depth. p53 is a critical molecule in doxorubicin-mediated apoptosis as it activates several molecules involved in cell cycle arrest and apoptosis, including p21, NF-κΒ, and STAT3. Thus, Hsp27-mediated inhibition of p53 results in the downregulation of all these pathways and the subsequent inhibition of apoptosis [86]. However, Xu et al. found that the phosphorylation status of Hsp27, rather than the total amount of Hsp27, is linked with the activation of p53. Specifically, they observed that the doxorubicin-induced DNA damage caused the activation of ATM, which resulted in the phosphorylation of Hsp27, subsequent activation of p53, and eventually cell cycle arrest, whereas the dephosphorylated form inactivated p53 and promoted cell cycle progression [32].

**Figure 5:**
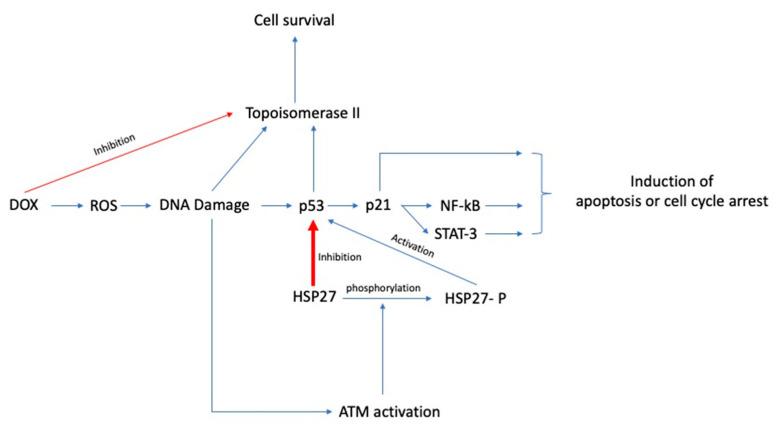
Summary of the proposed mechanisms by which Hsp27 induces doxorubicin chemoresistance. Hsp27 overexpression or overactivation results in inhibition of p53, a key molecule involved in doxorubicin induced apoptosis and cell cycle arrest. Key: DOX, doxorubicin; ROS, reactive oxygen species; STAT3, signal transducer and activator of transcription 3; NF-kb, nuclear factor kappa-light-chain-enhancer of activated B cells; ATM, ataxia-telangiectasia mutated kinase.

In another in vitro study, there was attenuation of doxorubicin resistance when cancer cells were treated with imatinib, a tyrosine-protein kinase inhibitor. Imatinib reverses both the intrinsic and acquired resistance to doxorubicin in cancer cells with highly active tyrosine-protein kinase c-Abl, a kinase overactivated in several metastatic solid organ cancers and blood malignancies. Contrary to the aforementioned studies suggesting a P-gp independent mechanism of doxorubicin resistance, the authors found that imatinib directly inhibits P-gp. STAT3 is a transcription factor usually overactivated in several advanced malignancies and involved in cellular survival, invasiveness, angiogenesis, and drug resistance. STAT3 has a critical role in the development of doxorubicin resistance. Specifically, it appears to promote the doxorubicin-mediated intranuclear translocation of NF-kb/p65, thus repressing both the antiapoptotic protein expression and the activation of the Hsp27/p38/Akt survival pathway. Imatinib directly inhibits STAT3 and the activation of the aforementioned pathways, eventually reversing doxorubicin resistance [84]. Similarly, Zhang et al. mentioned that the doxorubicin resistance in the MCF-7 breast cancer cell line was reversed after the knockdown of endoplasmic reticulum protein 29 (ERp29), a reticuloplasmin involved in endoplasmic reticulum proteostasis and protein excretion. In particular, they observed that ERp29 induced the overexpression of Hsp27 by downregulating the expression of eukaryotic translational initiation factor 2α (eIF2α), while the knockdown of Hsp27 in MDA-MB-231 breast cancer cells overexpressing ERp29, attenuated the doxorubicin resistance [87]. The authors concluded that eIF2a downregulates Hsp27 expression at a translational level, but the exact mechanism remains unclear. In response to ER stress eIF2a is phosphorylated by PKR-like ER kinase (PERK), and its phosphorylation results in cell cycle arrest at the G1 phase [88]. However, the authors reported that the relative phosphorylation of eIF2a was not significantly different between ERp29 overexpressing cells and control, while the basal expression of eIF2a was significantly decreased, at approximately 2.5-fold. Hence, the latter data indicate that the ERp29 inhibits eIF2a via decreasing its expression levels, rather than being involved in its phosphoregulation. Moreover, Hsp27 seems to have a cytoprotective role in the castrate-resistant prostate cancer cell line (LNCaP) by directly chaperoning the eukaryotic translation initiation factor 4E (eIF4E) and protecting it by ubiquitination, consequently promoting cell growth and survival [89]. In contrast, Kanagabasai et al. found that Hsp27 overexpression resulted in the reversal of doxorubicin resistance in doxorubicin-resistant MCF-7 breast cancer cells by downregulating MDR1/P-gp expression. The authors concluded that the inhibition of Hsp27, which typically results in augmentation of proteasomal p53 degradation, is inhibited in the doxorubicin-resistant MCF-7 cell line, resulting in intracellular p53 accumulation and increased nuclear factor NF-kB dependent MDR1/P-gp expression. The forced expression of Hsp27 by transcriptional activation of Hsp27 resulted in the reversal of doxorubicin resistance [90]. Mahvi et al. found that toremifene, an antiestrogen with a similar structure to tamoxifen, can also reduce the doxorubicin resistance in Hsp27-overexpressing MDA-MD-231 breast cancer cell lines and induce a cell cycle arrest at the G2/M phase of the cell cycle, but the mechanism of action was not investigated [91]. A summary of the proposed mechanisms of Hsp27-induced doxorubicin chemoresistance are presented in Figure 5.

### 3.2. Herceptin/Trastuzumab

Kang et al. reported that Hsp27 is probably involved in the resistance of breast cancer cells to herceptin (trastuzumab) [92]. Herceptin is a monoclonal antibody that targets HER2 receptors in HER2-overexpressing cancers, such as in some subtypes of breast and gastric cancers. HER2 is a transmembrane receptor tyrosine kinase that activates several important cell survival and proliferation intracellular signaling pathways such as PI3/Akt and Ras/RAF/MEK/ERK. Amplification or overactivation of the HER2/Neu oncogene, the gene that encodes HER2, is observed in approximately 30% of all breast cancers, the so-called HER2(+) breast cancers. However, most patients treated with Herceptin develop resistance after the first year of treatment. In their in vitro study, the authors isolated a subtype of SK-BR-3 breast cancers with resistance to Herceptin, the SK-BR-3-HR. The SK-BR-3-HR cells expressed high levels of Hsp27, while the downregulation of Hsp27 with siRNA resulted in the reversal of doxorubicin resistance. With immunochemistry studies, the authors concluded that Hsp27 binds to HER2 and increases its stability [92]. Hwang et al. found that the phosphorylation of Hsp27 at S15 residue is a critical event in the induction of the aforementioned Hsp27 mediated resistance to herceptin. Specifically, the S15 phosphorylation promotes the intranuclear localization of Hsp27 and subsequently the activation of the AKT survival pathway. J2 is a functional inhibitor of Hsp27 that impairs the dimerization of Hsp27 and phosphorylation at the S15 residue and inhibits the intranuclear localization of Hsp27, ultimately leading to herceptin sensitization [93].

### 3.3. Gemcitabine

Gemcitabine is a synthetic pyrimidine analog utilized as a first-line treatment for pancreatic cancer, exerting its anticancer effect through induction of apoptosis and inhibition of DNA synthesis in cancer cells. However, most patients with pancreatic cancer develop resistance to gemcitabine, ultimately leading to poor patient outcomes [94]. Currently, there is mounting evidence from proteomic and transcriptomic studies that the overexpression of Hsp27 is profoundly involved in the gemcitabine resistance to pancreatic cancer whereas, the downregulation of Hsp27, by siRNAs or other molecules that directly inhibit the Hsp27 activity [95], restores the sensitivity to gemcitabine in pancreatic cancer cell lines [96,97,98,99,100,101,102,103,104]. Zhang et al., in their in vitro study [96], utilized SW1990/gemcitabine-sensitive pancreatic cells and SW1990/gemcitabine-resistant pancreatic cells. Compared to the former, the latter cell line showed a significant increase in Hsp27 expression, along with overexpression of Snail, a DNA-binding zinc finger protein and transcriptional repressor of E-cadherin, which is also involved in epithelial to mesenchymal migration of cancer cells. Both cell lines were subsequently transfected with Hsp27 small hairpin RNA (shRNA) which downregulates the Hsp27 expression. This downregulation of Hsp27 led to induction of apoptosis, decrease in Snail and ERCC1, and an increase in E-cadherin levels. Although the exact molecular mechanisms were not clarified, the authors suggested that Hsp27-mediated upregulation of Snail resulted in cytoskeletal changes such as a decrease in E-cadherin, eventually promoting the EMT of pancreatic cancer cells, while the increased expression of ERCC1 has been associated with nucleotide excision repair after chemotherapy [51,98,105]. In another in vitro study, Kang et al. used three different pancreatic cell lines resistant to gemcitabine: HPAC, MiaPaCa-2, and BxPC3. Hsp27 downregulation by recombinant adenovirus expressing TRAIL and Hsp27 shRNA resulted in reduced cell viability in K-ras mutated pancreatic cells including HPAC and MiaPaCa-2 after treatment with gemcitabine. However, this cancer cell death was necrotic and not apoptotic, as suggested by the increase in the necrotic marker extracellular high mobility group 1 (HMGB1). Of note, the authors proposed that the ratio of phosphorylated to non-phosphorylated Hsp27 is the most determinant factor to predict gemcitabine resistance. Additionally, they suggested that the gemcitabine-induced p38/MAPK activation, which leads to phosphorylation of Hsp27, may eventually lead to Hsp27-CHK1 interaction [103]. CHK1 is a serine/threonine kinase that, under normal cell conditions, regulates DNA replication in the S phase, progression from the G2 phase to mitosis and mitotic exit, whereas under cell stress interacts with other kinases, delaying the cell cycle progression, thus allowing time for DNA damage repair [106]. Consequently, the Hsp27-CDK1 interaction presumably leads to CDK1 activation, more time for repairing gemcitabine-induced DNA damage, and eventually increased chemoresistance [103]. Nakashima et al. demonstrated that gemcitabine can induce activation of p38 mitogen-activated protein kinase (MAPK) and MAPK-activated protein kinase 2 (MAPKAPK-2) in the Panc1 pancreatic cancer cell line, and the activation of these kinases results in the phosphorylation of Hsp27 at Ser15, 78, and 82 residues. In order to elucidate the role of the phosphorylated Hsp27, the authors created two types of mutant HSP27-transfected Panc1 cells: 3A cells overexpressing non-phosphorylatable Hsp27 and 3D cells mimicking overexpressing Hsp27 Panc-1 cells. After treatment with gemcitabine, 3D cells demonstrated a significant attenuation of tumor cell growth compared to 3A cells, indicating that the phosphorylated Hsp27 promotes gemcitabine-induced tumor growth suppression [104].

**Figure 6:**
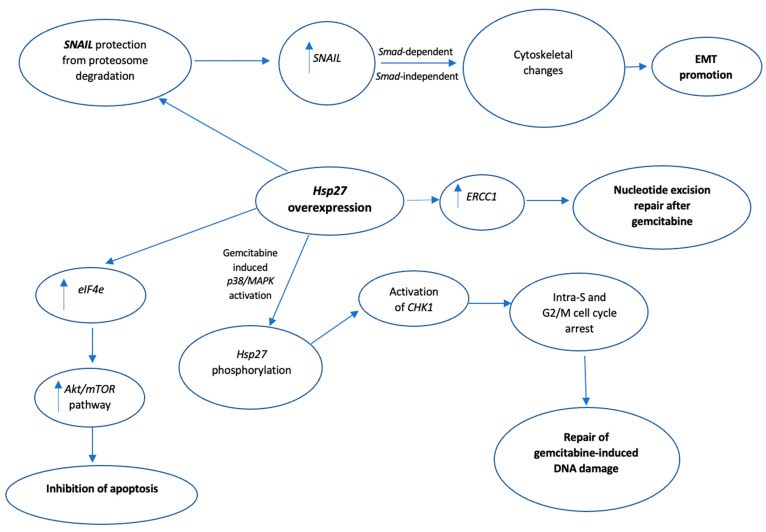
Summary of the proposed mechanisms by which Hsp27 mediates gemcitabine chemoresistance. Hsp27 overexpression or phosphorylation, through different molecular pathways, results in inhibition of apoptosis, promotion of EMT and repair of gemcitabine-induced DNA damage. Key: SNAIL, zinc finger protein SNAI1; EMT, epithelial to mesenchymal transition; ERCC1, effects of excision repair cross-complementation group 1; mTOR, mammalian target of rapamycin; CHK1, checkpoint kinase 1; eIF4e, eukaryotic translation initiation factor 4E.

In a novel study, Baylot et al. found that OGX-427, an antisense oligonucleotide that works as an HSP27 inhibitor, can increase the gemcitabine sensitivity in the pancreatic cancer MiaPaCa-2 cell line via decreasing the expression of eukaryotic translational initiation factor 4E (eIF4E), a downstream molecule of the mTOR pathway upregulated in numerous cancers and associated with tumor aggressiveness and chemoresistance [107]. The latter observation indicated a new molecular mechanism of Hsp27’s cytoprotective role against gemcitabine, involving upregulation of the Akt/mTOR signaling pathway and subsequent inhibition of cancer cell apoptosis. In vivo, the combination of OGX-27 and gemcitabine reduced the Hsp27 levels and sensitized mice’s tumors to gemcitabine. Mori-Iwamoto et al. utilized two distinct pancreatic cell lines: gemcitabine-sensitive KLM1 cells and gemcitabine-resistant KLM1-R cells. On the latter occasion, there was Hsp27 upregulation. Additionally, downregulating Hsp27 expression in the KLM1-R cell line through treatment with interferon-γ (IFN-γ) resulted in increased gemcitabine sensitivity compared to normal KLM1-R cells, control KLM1-R cells, and normal KLM1 cells [108]. Other molecules that decreased the expression of HSP27 in gemcitabine resistance pancreatic cancer cells included the basidiomycete mushroom active hexose-correlated compound (AHCC) [101], the N-formyl-3,4-methylenedioxy-benzylidene-γbutyrolactam (KNK437) [109], and the extract of Tripterygium wilfordii, a traditional Chinese herb, triptolide [110]. However, the exact mechanism of Hsp27 inhibition by these molecules is not clear. A summary of the proposed mechanisms by which Hsp27 mediates gemcitabine chemoresistance are presented in Figure 6.

### 3.4. 5-FU

**Figure 7:**
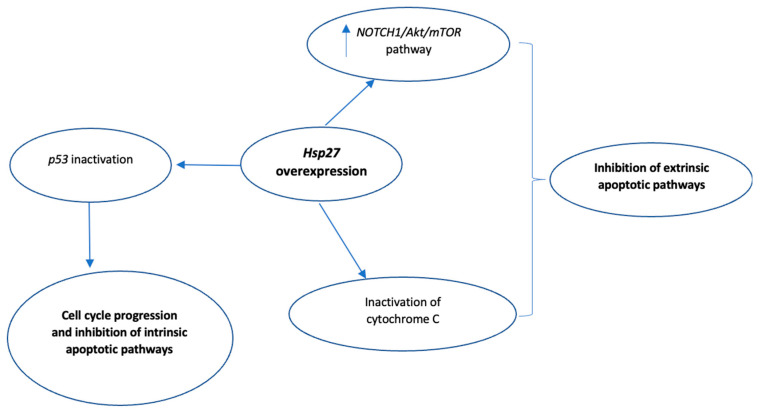
Summary of the molecular mechanisms of Hsp27-mediated resistance against 5-Fluorouracil (5-FU). Inhibition of both intrinsic and extrinsic apoptotic pathways play a key role in the 5-FU resistance. Key: NOTCH1, notch homolog 1; mTOR, mammalian target of rapamycin.

Currently, there is mounting evidence that Hsp27 is also involved in the chemoresistance of colorectal cancer cells to 5-Fluorouracil (5-FU) [32]. Broadly utilized as a chemotherapeutic agent in multiple types of cancer, including several gastrointestinal tract cancers, as well as breast and lung cancers, 5-FU is a thymidylate kinase inhibitor blocking DNA replication. It exerts its anticancer effect through various molecular mechanisms, including induction of apoptosis and cancer cell growth arrest [111,112]. Apoptosis induced by 5-FU is mediated primarily through the downregulation of the Akt/mTOR signaling pathway, a negative regulator of apoptosis, while the cancer cell growth inhibition is the result of the 5-FU-induced p53 activation, which in turn leads to G1/S cell cycle arrest [112]. Liu et al. found that SW480 colon cancer cells, which had been transfected with a lentivirus vector containing shHsp27 (short hairpin RNA molecules downregulating Hsp27), had increased sensitivity to 5-FU compared to control cells (transfected with an empty lentivirus vector) [111]. The latter increased chemosensitivity, due to induction of apoptosis, was mediated by Hsp27 suppression which resulted in a decrease in NOTCH-1 expression and subsequent suppression of the AKT-mTOR pathway, thus acting synergistically with 5-FU. These results were also confirmed in vivo in mice xenografted with SW480 colon cancer cells [111]. In another study, HT-29 and LoVo colon cancer cell lines were cultured with high doses of 5-FU to become 5-FU-resistant. Compared to parental colon cancer cells, the 5-FU-resistant colon cancer cells demonstrated downregulation of MiR-214, a small non-coding RNA that has been found to act as a tumor suppressor in colon cancer cells. When the resistant colon cancer cells were transfected with miR-214 mimics, thus leading to MiR-214 overexpression, these cells exhibited increased cell growth inhibition of 5-FU. This indicated that miR-214 sensitizes colon cancer cells to 5-FU. Of note, the authors observed that this sensitization is the result of MiR-214 interaction with Hsp27, specifically by binding to the 3′ UTR of Hsp27 and thus downregulating it [113]. Similarly, Jiang et al. suggested that another microRNA, MiR-577, also had tumor suppressor properties in 5-FU-resistant SW480 colon cancer cells through direct MiR-577 and Hsp27 interaction and subsequent induction of G0/G1 cell cycle arrest, yet the exact molecular mechanisms behind this cell cycle arrest were not described. These results were also validated in vivo as mice xenografted with MiR-577-overexpressing SW480 cells experienced attenuation of tumor growth compared to control tumors [114]. In another study, Sharma et al. found that the combination of 5-FU and carboplatin increases the expression of Hsp27 and Hsp40 in Hep3B and HepG2 hepatoma cell lines. According to the authors, this 5-FU-induced overexpression of Hsp27 helps cells survive the cytotoxicity of the drug. The latter was confirmed by further in vitro studies in which Hsp27 was downregulated either with quercetin (Hsp inhibitor) or with Hsp27-specific siRNA, which led to Hsp27 downregulation and subsequently enhanced cytotoxicity of 5-FU in both hepatoma cell lines. This cytotoxicity involved G1/S cell cycle arrest, presumably due to the inactivation of Hsp27 (and consequently activation of p53), thus p53-mediated G1/S cell cycle arrest [86], as well as induction of apoptosis attributed to the lack of interaction between Hsp27 and cytochrome C [115]. A summary of the molecular mechanisms of Hsp27-mediated chemotherapy resistance against 5-FU are presented in Figure 7.

### 3.5. Temozolomide

Temozolomide (TMZ) is an oral alkylating agent which, in combination with radiotherapy, has been found to promote overall survival in glioma patients. TMZ exerts its anticancer properties through the formation of O6-methylguanine in DNA that mis-pairs with thymine during DNA replication, resulting in G2/M phase cell cycle arrest and eventually cell death by apoptosis or autophagy [116,117].

Quercetin (3,3′,4′,5,7-pentahydroxyflavone) is a flavonoid found in fruit and vegetables associated with various anticancer effects, including reduction in cancer resistance in human glioblastoma cell lines, leukemia, and oral cancers [118,119,120,121,122,123,124]. In U251 and U87 human glioblastoma (GBM) cell lines, quercetin was involved in the TMZ-mediated apoptosis, as suggested by the increased activity levels of caspase 3. In particular, the combination of quercetin and TMZ exerted a better cytotoxic effect in vitro compared to TMZ alone. Using Hsp27-specific siRNAs, the authors proposed that this chemosensitization was due to the quercetin-induced Hsp27 inhibition, which in turn led to the promotion of mitochondrial apoptotic pathways, eventually enhancing the apoptotic effects of TMZ [118]. Li et al., using U251 and U87 GBM cell lines in vitro, found that treating these cells with t-AUCB, a soluble epoxide hydrolase inhibitor with antitumor properties, induced cell cycle arrest in the G1 phase. The authors also reported that the treatment with t-AUCB led to Hsp27 activation, which in turn enhanced COX-2 expression, a key inflammation marker associated with poor prognosis in gliomas. However, when the GBM cells were first treated with quercetin, which blocks both Hsp27 and COX-2, the combination of t-AUCB and quercetin displayed better antiproliferative effects compared to monotherapy with t-AUCB, suggesting a significant role of Hsp27 and COX-2 in t-AUCB resistance. However, the molecular aspects of the Hsp27- and COX-2-mediated t-AUCB resistance were not clarified. The sensitization of GBM cells to t-AUCB with the use of quercetin was also observed in vivo in mice xenografted with U87 GBM cells [121]. In another study, the same authors, using the same GBM cell lines in vitro, reported that t-AUCB enhanced the expression of Atg7, a downstream molecule of Hsp27 which participates not only in autophagosome formation but also in the apoptotic resistance of GBM cells to t-AUCB. Additionally, the combination of quercetin and t-AUCB had a better antiproliferative effect compared to t-AUCB alone, attributed to quercetin-induced blockage of Atg7-mediated autophagy [122]. Jakubowicz-Gil et al., using T98G human GBM cells in vitro, reported that quercetin increased the TMZ-induced ER stress and facilitated the TMZ-induced apoptosis. Both were the result of quercetin-mediated Hsp27 blockage, yet the exact molecular mechanisms remained unknown [123]. The induction of apoptosis, as indicated by the increased activity of caspase-3 and caspase-9, was the main cytotoxic mechanism of the quercetin and TMZ drug combination not only in T98G GBM cells but also in human brain astrocytoma MOGGCCM cells. Once again, the drug combination did not affect the induction of autophagy [124].

Although the aforementioned studies did not provide evidence regarding the molecular aspects of Hsp27 mediated cytoprotection against temozolomide, there are in vitro studies suggesting that Hsp27 overexpression increases the expression of Akt and decreases ROS levels [125]. The former results in upregulation of the Akt/mTOR signaling pathway and subsequent inhibition of apoptosis, while the latter effect leads to suppression of intrinsic mitochondrial apoptotic pathways [126].

### 3.6. Paclitaxel

Paclitaxel is a chemotherapy drug frequently utilized for the treatment of various cancers, including ovarian, breast, and lung cancer. Regarding its mechanism of action, paclitaxel binds to the microtubule protein β-tubulin, and stabilizes the mitotic spindle, ultimately leading to the inhibition of mitosis and cell proliferation. Although the drug is commonly utilized in the treatment of ovarian cancer, the development of resistance to it is not unusual, eventually leading to poor outcomes. Hsp27 is overexpressed in aggressive forms of ovarian cancer and seems to be involved in that paclitaxel resistance. An inhibitory effect of paclitaxel in the expression of Hsp27 was observed in studies using ovarian cancer cell lines in vitro [127]. Except for binding to microtubules, paclitaxel promotes the formation of free radicals and the induction of cellular oxidative stress, probably by activating the NADPH oxidase. Hence, it is reasonable to assume that the overexpression of Hsp27, an oxidative stress-protective agent, may limit the effectiveness of paclitaxel. It was reported that the downregulation of Hsp27 by small interfering RNAs (siRNAs) and the consequent inhibition of its chaperoning activity in oxidative stress seem to enhance the sensitivity of paclitaxel in human ovarian cancer cells (HO8910) as well as in bladder cancer cells by increasing apoptosis rate [128,129]. The involvement of free radicals in the latter effect is enhanced by the fact that the use of N-acetyl-cysteine, a ROS scavenger, limits the effect of si-RNA-mediated Hsp27 silencing [128]. In human bladder cancer UMUC-3 cell in vitro, the Hsp27 downregulation via siRNA or OGX-427 (an antisense oligonucleotide targeting Hsp27) results in the inhibition of cancer cells growth and an increase in the apoptotic rate after treatment with paclitaxel. UMUC-3 has a mutant p53 gene, therefore the mechanism of apoptosis inhibition is probably p53-independent, and the result is probably mediated through a decrease in the activation of caspase-3. OGX-427 seems to enhance the efficacy of paclitaxel in vitro and the apoptotic rate in vivo in UMUC-3-xenografted mice [129]. Regarding the effects of docetaxel (another taxane) in the development of drug resistance, Stope et al. found that it induced the activation of mitogen-activated protein kinase p38 (p38 MAPK) and protein kinase D1 (PKD1) in prostate cancer cells. The activation of these kinases resulted in a rapid yet transient (approximately 48-h duration) phosphorylation of Hsp27 associated with increased sensitivity against docetaxel, while the permanent phosphorylation of Hsp27 resulted in an increased docetaxel resistance [130].

## 4. Other Hsp27 Inhibitors

RP101 (Brivudine) is a nucleoside that directly interacts with Hsp27 through binding π-stacking with Phe33 and Phe29 of Hsp27, ultimately attenuating the Hsp27-mediated promotion of apoptosis [1]. In rat sarcoma cells AH13r, the combination of RP101 with mitomycin C achieved better control of cell proliferation compared to mitomycin C alone, while in human fibrosarcoma cells HT-1080, gemcitabine, and RP101 decreased tumor invasiveness by approximately 40% in comparison to gemcitabine alone. In vivo, RP101 together with cyclophosphamide or cisplatin exerted superior antiproliferative effects compared to monotherapy in Sprague–Dawley (SD)-rats xenografted with AH13r sarcoma cells [131]. Furthermore, in a pilot clinical study in patients with pancreatic cancer, the combination of RP101 and gemcitabine produced a survival benefit of 8.5 months compared to gemcitabine alone, but in a phase II clinical trial, the combination therapy increased median survival by around 2.20 months compared to monotherapy with some gemcitabine-induced adverse effects, particularly in lightweight patients [131].

Ivermectin is an antiparasitic drug utilized for the treatment of several parasites such as in infection by threadworms (Strongyloides stercoralis), onchocerca volvulus (river blindness), filariasis, and scabies. In a novel study, Nappi et al. found that ivermectin inhibits the MAPKAPK2-mediated phosphorylation of Hsp27 in serine residues at the phosphorylation pockets of Hsp27’s 24-mers that are formed by twelve Hsp27 dimers. The phosphorylation of these serine residues under normal circumstances results in the depolymerization of 24-mers and the subsequent activation of several signaling pathways. Hence, ivermectin impairs the phosphoregulation of Hsp27 and results in the downregulation of these signaling pathways, including EGFR and HER2. In their study, they also reported that Hsp27 inhibits phosphatase SHPTP1, which dephosphorylates and activates EGFR. Erlotinib, an EGFR inhibitor utilized in lung cancer, can induce an increase in Hsp27 activation and subsequent inhibition of SHPTP1, which can limit its effectiveness. Ivermectin impaired the Hsp27-mediated inhibition of SHPTP1 and reduced the resistance to erlotinib. Ivermectin also sensitized castrate-resistant prostate cancer cells deprivation therapy, probably by impairing the Hsp27 mediated nuclear transportation of androgen receptors [132]. Table 1 summarizes the Hsp27 inhibitors, the chemotherapy agents whose activity was enhanced, and each cancer type.

## 5. Clinical Trials with Hsp27 Inhibitors

Currently, only a few clinical trials have studied whether Hsp27 inhibitors can enhance the efficacy of the established chemotherapy agents in humans [131,133,134]. In a phase I study in 42 patients with castrate-resistant prostate cancer, the agent OGX-427 decreased the number of tumor circulating cells and prostate-specific antigen levels (the latter was reduced by over 50% in 10% of the patients) [133]. In a multicenter phase II clinical trial (“Borealis”) with 99 patients, the combination of OGX-427 and docetaxel improved the overall survival of patients with metastatic urothelial cancer compared to docetaxel alone. Interestingly, patients with more favorable outcomes demonstrated lower Hsp27 levels [134]. Conversely, in another phase II trial with non-squamous cell carcinoma, OGX-427 did not improve the overall survival in patients treated with the standard therapy with carboplatin [135]. Similarly, in the RAINIER phase II trial in patients with metastatic pancreatic cancer, OGX-427 did not improve the overall survival of patients treated with OGX-427 and gemcitabine/paclitaxel versus the standard therapy. However, the patients with a more favorable prognosis displayed lower Hsp27 levels [136]. Regarding the other Hsp27 inhibitors, only brivudine has been evaluated in a phase II clinical trial in pancreatic cancer patients (discussed in Section 4) [132].

## 6. Hsp27 as a Potential Biomarker

Hsp27 overexpression has been described as an indicator of poor outcomes in patients with various cancer types including, non-small lung cancer, breast cancer, colorectal cancer, melanoma, glioma, prostate cancer, intrahepatic cholangiocarcinoma and hepatocellular carcinoma [27,28,29,30,31,32,34,41,42,63,79,137,138,139]. Specifically, Hsp27 was correlated with tumor size, TNM stage, degree of differentiation, and histological subtype [30,32,137]. Conversely, there are also cancer types, such as neck and head squamous cell carcinoma [33] and neuroblastoma [140], where low levels of Hsp27 are associated with worse outcomes. Consequently, there is an inconsistency among the various cancer types as to whether the high levels or the low levels of Hsp27 could indicate poor clinical outcomes. Additionally, most of the aforementioned data derive from retrospective studies. As a result, Hsp27 is not an established cancer biomarker for the time being; nevertheless, through prospective studies and clinical trials, it has the potential to become one in the future.

## 7. Conclusions

Hsp27 is associated with the development of resistance against multiple chemotherapy agents, in various cancer types, and by different molecular mechanisms, while the downregulation or inhibition of Hsp27 leads to the reversion of resistance. However, the exact mechanisms of Hsp27-mediated resistance remain unclear and vary in different types of cancer, thus necessitating the conduction of further studies to clarify them. The Hsp27-mediated inhibition of the tumor suppressor p53 gene by a currently unknown mechanism is linked with the resistance to doxorubicin probably via decreased activation of p53-downstream targets such as p21 and STAT3 that are involved with cellular senescence and apoptosis induction, respectively. Moreover, the inhibition of p53 probably decreases the activation of topoisomerase II, a primary doxorubicin target, and hence the Hsp27-dependent p53 inhibition may result in increased doxorubicin resistance. On the contrary, the cytoprotective effect of Hsp27 from paclitaxel-induced cellular stress seems to be mediated via a p53-independent mechanism. Finally, the data regarding the role of Hsp27 in herceptin resistance remain in their infancy, but Hsp27 seems to bind and stabilize the HER2 receptor. Apart from p53 modulation, another common mechanism of Hsp27-induced chemoresistance is the upregulation of the Akt/mTOR signaling cascade which eventually leads to the inhibition of cancer cell apoptosis. The latter was a key molecular mechanism of chemoresistance in at least three different chemotherapeutic agents (gemcitabine, 5-FU and temozolomide). The proposed mechanism by which the Hsp27 mediated chemoresistance is induced is summarized in Figure 2. A number of molecules, including RP101 (Brivudine) and Quercetin, and OGX-427 have yielded promising results in vitro, in vivo as well as in early-stage clinical trials, thus not only confirming the key role of Hsp27 in cancer’s chemotherapy resistance but also indicating a novel therapeutic approach.

## Figures and Tables

**Table 1:** Summary of Hsp27 modulators that can be utilized as potential chemotherapy sensitizers.

Sensitizer	Chemotherapy Agent	Cancer Type	Reference
Paclitaxel	Doxorubicin	Breast cancer	[82]
Imatinib	Doxorubicin	Colorectal	[84]
OGX-427	Gemcitabine	Pancreatic	[107]
	Erlotinib	Lung	[12]
AHCCC	Gemcitabine	Pancreatic	[101]
KNK437	Gemcitabine	Pancreatic	[109]
Triptolide	Gemcitabine	Pancreatic	[110]
Quercetin	5-FU	Hepatoma	[115]
	TMZ	GBM	[118]
	t-AUCB	GBM	[121]
Toremifene	Doxorubicin	Breast cancer	[91]
BVDU	Bortezomib	Multiple myeloma	[131]

Key: Hsp27, heat shock protein 27; AHCCC, active hexose-correlated compound; KNK437, N-formy1–3,4-methylenedioxy-benzylidene-γbutyrolactam, 5-FU, 5-Fluorouracil; TMZ, temozolomide; GBM, glioblastoma.

## Data Availability

Not applicable.

## Abbreviations

Hsp27, heat shock protein 27; MAPK, P38 mitogen-activated protein kinase; SWH, Salvador-Warts-Hippo; YAP/TAZ, Yes-associated protein/transcriptional coactivator with PDZ-binding motif; EMT, epithelial to mesenchymal transition; NF-κB, nuclear factor kappa-light-chain-enhancer of activated B cells; TGF-β1, transforming growth factor beta 1; TLR3, toll-like receptor 3; VEGF, vascular endothelial growth factor; STAT3, signal transducer and activator of transcription 3; ER, endoplasmic reticulum; COX, cyclooxygenase; HER, human epidermal growth factor; mTOR, mammalian target of rapamycin; CSCs, cancer stem cells; JNK, c-Jun N-terminal kinase; ERK, extracellular signal-regulated kinase; MDR, multidrug resistance; p38 MAPK, mitogen-activated protein kinase p38; GBM, glioblastoma; eIF, eukaryotic translational initiation factor; TMZ, temozolomide; siRNA, small interfering RNA; shRNA, small hairpin RNA; EGFR, Epidermal growth factor receptor tyrosine kinase; 5-FU, 5-Fluorouracil; AHCCC, Active hexose-correlated compound; KNK437, N-formyl1–3,4-methylenedioxy-benzylidene-γbutyrolactam; NADPH, nicotinamide adenine dinucleotide phosphate; IFN-γ, interferon-γ; ROS, reactive oxygen species; ER, endoplasmic recticulum; HMGB1, extracellular high mobility group 1; Chk1, checkpoint kinase 1; KNK437, N-formyl-3,4-methylenedioxy-benzylidene-γbutyrolactam; 3′ UTR, three prime untranslated region; PI3K, phosphoinositide 3-kinases.
